# Influence of *CYP2B6* Genotype on Methadone Dosage in Patients from the Methadone Maintenance Treatment (MMT) Program in Pereira, Colombia

**DOI:** 10.3390/life13041038

**Published:** 2023-04-18

**Authors:** Carlos Isaza, Oscar Mauricio Castaño-Ramírez, Juan Pablo Vélez, Julieta Henao, Leonardo Beltrán-Angarita, Juan Carlos Sepúlveda-Arias

**Affiliations:** 1 Facultad de Ciencias de la Salud, Universidad Tecnológica de Pereira, Pereira 660003, Colombia; 2 Facultad de Ciencias de la Salud, Universidad de Caldas, Manizales 170004, Colombia; 3 ESE Hospital Mental de Risaralda, Pereira 660001, Colombia; 4 Grupo Infección e Inmunidad, Facultad de Ciencias de la Salud, Universidad Tecnológica de Pereira, Pereira 660003, Colombia

**Keywords:** methadone, heroin, pharmacogenetics, pharmacodependence, *CYP2B6*, Colombia

## Abstract

Methadone treatment reduces the use of heroin and withdrawal symptoms; however, methadone is an expensive medication with a narrow safety margin. We compared the retention rates, persistence of heroin use, and quality of life of a group of patients undergoing conventional Methadone Maintenance Treatment (MMT) with a group for whom the *CYP2B6* 516G>T polymorphism was used in addition to the MMT to calculate the required methadone dose. Over 12 weeks, the retention rate, heroin usage, and quality of life of patients under conventional treatment (n = 34) were compared with those of patients for whom we used genetic markers to calculate methadone dosage (n = 38). At the end of the study, 26.4% of patients abandoned the program, and neither demographic nor clinical variables were associated with treatment adherence. Of the remaining patients, 16% of the control group and 8% of patients in the pharmacogenetic group reported heroin use, while both groups showed a 64% reduction in the use of cocaine/crack (no significant differences between the groups were found). Starting in the second week, the methadone dosage was lower among the patients for whom methadone was prescribed based on genotype. Although there were six individuals in the control group and three in the pharmacogenetic group with QTc intervals > 450 ms (a threshold that is considered dangerous), we did not find a relationship between the QTc interval and methadone dosage. There were no differences in the perception of quality of life between the two groups. The results of this pilot study suggest that concerning methadone therapy, the *CYP2B6* genotype contributes to reduced effective doses and treatment costs.

## 1. Introduction

As seen in several developing countries, the problem of psychoactive drugs in Colombia is significant given that the production, exportation, importation, and consumption of illegal drugs all happen in this country. This situation has overwhelmed the public health system and has resulted in corruption and violence derived from illegal drug sales. According to official data, in the last six years, there has been an overall increase in the use of illicit drugs (marijuana, cocaine, crack, ecstasy, and heroin), and it is estimated that 350,000 individuals will require some form of treatment for drug abuse and dependency [1].

According to the World Health Organization, alcohol, tobacco, and heroin are problematic substances that cause the heaviest burden of disease and drug-related deaths worldwide [2]. One out of four people who use heroin for the first time become drug dependent. Among these individuals, mortality is 10–20 times higher than among non-drug users (paired by age and gender) and 12 times higher than in the general population [3]. In addition, heroin-addicted patients often resort to crime and prostitution to finance their habit, adopting risky behaviors that translate into an increased risk of acquiring infectious diseases (hepatitis B and C, tuberculosis, HIV/AIDS, and sexually transmitted diseases) [4].

The heroin dependence treatment that has shown the most significant evidence of favorable results consists of both psychosocial support and methadone (Methadone Maintenance Treatment, MMT), a long-acting opioid agonist capable of blocking the euphoric effects of heroin, preventing abstinence syndrome, and reducing compulsive searching for the drug (craving). The efficacy of methadone is reflected in decreased heroin usage, reduced criminal behavior, improved cognitive function, and lower morbidity/mortality [5]. Nevertheless, there is broad inter-individual variability in response to methadone, and for a significant percentage of patients, the strategy of periodically adjusting the dose upward to reach the so-called anti-craving effect not only dangerously delays patient perception of improvement, but also carries significant risks of lack of efficacy and toxicity [6]. For this reason, even in better-structured treatment programs, patient retention rates are approximately 60% at six months [5].

Several genetic factors related to pharmacokinetics and pharmacodynamics can influence the effects of methadone and may be related to variations in individual responses to the drug. The CYP2B6 enzyme plays a dominant role in the metabolic inactivation of methadone [7,8]. It can easily be induced or inhibited by other drugs and is one of the most polymorphic of the *CYP* genes, with over 100 described SNPs, of which 16 are associated with increased, decreased, or no expression or activity of the enzyme [9,10,11]. The CYP2B6*6 variant is the most common of the variants under study and consists of changes in two nucleotides (*4: rs2279343, c785 A>G; and *9: rs3745274, c516G>T), resulting in reduced expression of the protein (a reduction in enzyme levels of 50–75%) due to a splicing site that is erroneously caused by SNP *9. It should be clarified that the nucleotides that constitute the *6 alleles also occur in isolation, although at a much lower frequency [12]. Many additional alleles have been described; however, they have a very low prevalence or appear to have a functional effect of limited relevance [13].

Although there is still no certainty about the effect of the *CYP2B6* gene polymorphism on the clinical effects of methadone, some findings are interesting: (i) compared with the carriers of *CYP2B6* genotype native *1/*1, the carriers of genotypes *1/*6 and *6/*6 have significantly decreased clearance of both S- and R-methadone; (ii) allele *6 carriers are slow metabolizers of methadone, particularly S-methadone, which is cardiotoxic; (iii) due in part to the higher proportion of allele *6 carriers among African and Hispanic individuals, methadone metabolism is slower in these ethnic groups than in Caucasians; (iv) the *CYP2B6* genotype can identify individuals who are at increased risk for methadone toxicity or potentially dangerous interactions with other drugs that are also substrates for the enzyme [11,13,14,15,16,17,18].

The growing problem of opioid abuse and the limited effectiveness of standard methadone dosage protocols based on a series of demographic and social variables justify the search for better treatment strategies with greater predictive powers to improve adherence rates and reduce relapses [19,20]. The aim of this study was to compare the retention rates, persistence of heroin use, and quality of life in a group of patients under conventional MMT with a group for whom the *CYP2B6* genotype was used in addition to MMT to calculate the required methadone dose.

## 2. Materials and Methods

### 2.1. General Considerations

Patients with a dependency on heroin who were under MMT treatment in the Drug Addict Attention Center of the Hospital Mental de Risaralda (HOMERIS, a public hospital that treats low-income people in the city of Pereira, Colombia) were enrolled in this pilot study. The patients were randomized to enter the control and the pharmacogenetic group using a previously constructed table of random numbers, according to the order of admission of the patients to the Methadone Maintenance Treatment (MMT).

### 2.2. Inclusion and Exclusion Criteria

The inclusion criteria were: (1) age between 17 and 40 years, (2) a diagnosis of opioid dependence based on the DSM IV criteria, (3) acceptance of the study requirements and provision of informed consent, and (4) no MMT within the previous six months. The following patients were excluded from the study: patients with brain trauma, chronic neurological disease, cognitive deficits, or severe psychiatric disease (dementia or psychosis) compromising their ability to sign the consent form, as well as patients with epilepsy, liver disease, kidney disease, CYP2B6 enzyme inducer or inhibitor use, pregnancy, or lactation. All individuals included in the study had severe levels of heroin dependence and lived in marginal socioeconomic conditions. Patients were not asked to abstain from consuming other drugs throughout treatment; however, during medical interviews and psychosocial interventions, efforts were made to control the use of other addictive substances.

### 2.3. Participants

Individuals were randomly assigned to one treatment group for 12 weeks. In the conventional protocol, patients were treated and dosed with methadone according to the criteria adopted by the MMT program in HOMERIS. This group comprised 34 unrelated individuals (29 men and 5 women with a mean age of 24.6 ± 5 years). In the pharmacogenetic protocol, patients were treated with the standard protocol with the addition of pharmacogenetics data to calculate methadone dosages. This group was composed of 38 unrelated individuals (34 men and 4 women with a mean age of 23 ± 4 years).

In both groups, methadone administration was supervised, and an initial dose of 40 mg/day was administered and periodically adjusted based on the following criteria: demographic characteristics, potential pharmacological interactions, self-reported craving behavior, self-reported symptoms of abstinence and impaired judgment, urine test results, and clinical findings of overdose. To monitor the risk of methadone cardiotoxicity (prolonged QT interval), control electrocardiograms were performed approximately one week after the medication was initiated [21]. To calculate methadone dosages in the pharmacogenetic group, the treating clinician was given one of the following recommendations according to the result of the *CYP2B6* genotype of the patient: (i) native homozygote: “adjust the dose based on clinical judgment and use high doses (≥80 mg/day) if necessary”; (ii) heterozygote *1/*6: “try to stabilize the patient with moderate doses (~60 mg/day)”; and (iii) mutated homozygote “the patient is a poor metabolizer; do not use doses higher than 40 mg/day”. The selection of the methadone dose was based on the *CYP2B6* genotype because the *CYP2B6* polymorphisms have been associated with methadone response and dose requirements in the setting of methadone maintenance therapy [22]. We also evaluated SNPs in the *ABCB1* and *OPRM1* genes because they are influential in the response to methadone. However, the analysis indicated that the SNPs evaluated in these two genes were monomorphic in our population [23] and did not influence the response, therefore we did not discuss them further throughout the study. Urine samples (one sample per week) were taken throughout the study without prior warning. The decision to adjust the methadone dose was the responsibility of the medical team based on program regulations.

Primary outcome measures included the following: (1) retention rates: the percentage of people who remained within the program throughout the study; and (2) reduction in the use of heroin, which was determined through self-reporting and/or periodic urine testing. Secondary outcome measures included the following: improved quality of life-based on the World Health Organization Quality of Life scale (WHOQOL-BREF), which estimates the quality of life in four areas: health and physical health, psychological health, social relationships, and environment [24]. The WHOQOL-BREF was evaluated through personal interviews in the eighth week of treatment. At the end of the study, patients retained the right to continue in the MMT program.

This pilot study was approved by the Ethics Committee of the Universidad Tecnológica de Pereira as minimum-risk research. An informed consent form was completed in accordance with Resolution 008430 of 1993 from the Ministry of Health of Colombia. The patient handling protocol was continuously adjusted according to MMT guidelines.

### 2.4. Paraclinical Tests

Tests for creatine, glutamic oxaloacetic transaminase (GTO), and glutamic pyruvic transaminase (GPT) were performed in an outsourced clinical laboratory. Electrocardiograms were obtained by a physician from the MMT program.

### 2.5. Genotyping

Genomic DNA was extracted from 200 µL of blood using the QIAamp Mini Kit, and genotyping was performed using a mini-sequencing method, as previously described [23]. An initial PCR was performed in a total volume of 10 µL containing 1–10 ng of DNA, 1X Qiagen Multiplex PCR Master Mix, and 0.2 µM of each primer (Table 1). The PCR procedure was as follows: an initial step of 15 min at 95 °C, followed by 35 amplification cycles at 94 °C for 1 min, 60 °C for 90 s, and 70 °C for 50 s, with a final extension at 72 °C for 7 min. The excess of primers and nucleotides was degraded by adding 2 µL of ExoSAP-IT (Affymetrix, Santa Clara, CA, USA) to 52 µL of the PCR product. Using this product as a template, multiplex reactions were performed to detect the SNPs in a volume of 6 µL, which contained 2 µL of PCR product, 2.5 µL of SNaPShot Reaction Mix (Applied Biosystems, Waltham, MA, USA), and 1.5 µL of SBE primer mix (Table 1). Amplification was performed with 35 cycles at 96 °C for 10 s, 50 °C for 5 s, and 60 °C for 30 s. For detection, 2 µL of this product was mixed with 9 µL of HiDi-formaldehyde and 0.5 µL of GeneScan-120Liz Internal Size Standard (Applied Biosystems) and read in an ABI Prims 3100-Avant Genetic Analyzer sequencer (Applied Biosystems) with a capillary of 36 cm and POP-4 polymer (Applied Biosystems). Data were analyzed based on the peaks’ colors and the fragments’ sizes using Genemapper V3.2 software (Applied Biosystems). The presence of the polymorphisms was confirmed by direct sequencing of the selected samples.

### 2.6. Statistical Analyses

After the normal distribution of the data was demonstrated (Kolmogorov-Smirnov test), a Chi-squared test was applied to compare the qualitative variables among the groups. Quantitative variables were compared using a Student’s *t*-test. All qualitative variables were dichotomous variables for the analyses. To evaluate the relationship between the methadone dose and the genotype of each patient, we used a regression model for repeated measures. The Kaplan-Meier estimate was used to compare the proportion of patients who remained in the program over time by following the “intention-to-treat” protocol, which included all patients who began the treatment. The analysis was performed with confidence intervals of 95%, and values of *p* < 0.05 were considered significant. Data were processed and analyzed via SPSS ver. 19 software for Windows.

## 3. Results

The Individuals included in this pilot study consisted of a young population (23.7 ± 4.8 years) that was predominantly male (87.5%) and had a low education level (49.3% did not complete secondary education). The majority were unemployed (69%), separated or single (91.5%), and had a criminal background (76%). The mean age of first heroin use was 18.7 ± 4 years, and the average duration of drug use was 5 ± 2.7 years. All individuals had previously taken psychoactive substances, and when entering the study, 90%, 70%, and 37.5% had a co-dependence on nicotine, marijuana, and cocaine/crack, respectively. Moreover, 81% of patients reported psychoactive substance abuse in a first-degree relative. All participants in the study displayed normal kidney and liver function test results.

Table 2 shows that there were no significant differences regarding the demographic and clinical characteristics of the patients in the group receiving conventional treatment and in the group for whom genetic variables were taken into account for methadone dosage.

The dropout rates in the program were 15.3% in the fourth week and 26.4% in the twelfth week. Retention rates in the MMT were established using the Kaplan-Meier method; the log-rank analysis showed no significant differences in treatment adherence (the percentage of people who remained within the program throughout the study) between the groups (*p* = 0.56). In the fourth week of treatment, none of the following variables affected the rates of methadone abandonment: age (*p* = 0.91), schooling (*p* = 0.05), marital status (*p* = 0.92), cohabitation (*p* = 0.38), current employment (*p* = 0.65), psychiatric disease history (*p* = 0.65), previous admittance to MMT (*p* = 0.34), age at which drug use began (*p* = 0.93), years of heroin abuse (*p* = 0.96), route of heroin administration (*p* = 0.53), co-dependency on cocaine (*p* = 0.21), co-dependency on marijuana (*p* = 0.57), family history of drug abuse (*p* = 0.66), and criminal background (*p* = 0.65). The lack of these associations was conserved throughout the study. No differences were observed in methadone doses among those who remained in the program at the end of the study and those who discontinued treatment in the control group (adherent 66 ± 20 mg/day vs. nonadherent 63 ± 10 mg/day, *p* = 0.71) or in the pharmacogenetic group (adherent 56 ± 12 mg/day vs. nonadherent 53 ± 16 mg/day, *p* = 0.63).

As shown in Table 3, heroin use (detected by self-reporting and/or urine testing) was reduced in both experimental groups beginning in the second week of treatment. This reduction was higher among individuals in the group in which pharmacogenetic variables were used; however, no significant differences between the groups were found. At the end of the study, 12% of patients had a positive urine heroin test. Regarding the effect of MMT on the abuse of other drugs, such as marijuana and cocaine/crack, it was found that in all cases, marijuana consumption remained at the same level throughout the study. In contrast, the proportion of individuals using cocaine/crack decreased from 37.5% to 13.5% at the final control (ninth week, 64% reduction). The groups had no differences in the use of both drugs at different time points (Table 4).

The genotype of each patient was correlated with two clinically relevant variables: the QTc interval and methadone dosage. Among a total of nine individuals (12.5%) with a QTc interval exceeding dangerous limits while taking methadone (>450 ms), six and three were in the control and pharmacogenetic groups, respectively. However, to evaluate the relationship between the methadone dose and the genotype of each patient, we used a regression model for repeated measures (406 records of weekly methadone doses for 72 patients). The analysis showed that the pharmacogenetics group received significantly lower methadone doses over the treatment period (*p* = 0.021) than the control group, with a high positive intraclass correlation coefficient (0.93).

The Hardy-Weinberg equilibrium was conserved in each of the three genotypes examined: *ABCB1* (rs1045642), *p* = 0.62; *CYP2B6* (rs3745274), *p* = 0.57; and *OPRM1* (rs1799971), *p* = 0.47. By exploring which genetic markers influenced methadone dosage, we found that only the polymorphism of the *CYP2B6* gene (rs3745274, 516G>T), which results in individuals being normal or low metabolizers, was related to the effective dose of the drug (Table 5). As indicated in the Materials and Methods section, the SNPs evaluated in the *ABCB1* and the *OPRM1* genes were monomorphic in our population and did not influence the response. The genotypic distribution of the polymorphism of the *CYP2B6* gene (rs3745274) was G/G 51.8%, G/T 36.4%, and T/T 11.8%.

The perception of quality of life according to the WHOQOL-BREF questionnaire, which was applied at the eighth week of treatment, was divided into three categories based on the grade of personal satisfaction: low (below the first quartile), medium (between the first and third quartile), and high (above the third quartile). Moreover, we determined if significant differences existed in any of the four domains assessed using the WHOQOL-BREF questionnaire. In both groups, the worst-qualified domain was social relationships (personal relationships, sex life, and support of friends), as only 9.8% of individuals were in the high quartile. Furthermore, this domain was associated with higher adherence rates and greater desertion among patients in the lower quartile than those in the medium and high quartile (*p* = 0.002). When the different parameters concerning quality of life were disaggregated by sex and compared, no differences were found between the groups.

## 4. Discussion

Demographic and clinical variables, as well as the start of heroin use and high co-dependency on other drugs of abuse (Table 2), are common features of patients admitted to rehabilitation and methadone maintenance programs. Methadone dosage began at 40 mg/day and was gradually increased until a maintenance dosage was reached. Although the overall risk of treatment dropout was similar in both groups of patients and the survival curves exhibited similar behavior, the low rates of abandonment of the program and the significant reduction in heroin consumption suggested that the standards of care for these MMT patients at HOMERIS were of high quality, making it difficult to identify differences between the groups given their sample sizes and the relatively short follow-up times. However, lower heroin consumption among individuals in the pharmacogenetic group should be highlighted, although such a trend was not significant (Table 3). The finding that cocaine/crack consumption was reduced collaterally by 64% in patients in the MMT is an interesting finding that has been previously reported [10]. This beneficial collateral effect can be attributed not only to the psychosocial recovery of patients, but also to the finding that methadone blocks glutamate receptors; in animal studies, antagonists of these receptors reduced the self-administration of cocaine and other psychoactive drugs [25].

Several studies detecting the demographic, clinical, and social predictive factors associated with the retention of heroin-addicted patients in different rehabilitation programs have yielded conflicting conclusions [26,27]. In this study, these variables did not affect the adherence of patients to the MMT, reaffirming the general opinion that both addictive behaviors and responses to treatment are complex psychosocial phenomena that are still poorly understood [26]. According to the Food and Drug Administration (FDA) in the United States, methadone is currently the drug most implicated in prolonging the ECG’s QT interval and inducing cardiac arrhythmias (Torsades de points, TdP). For this reason, it is recommended that patients receiving this medication be assessed to identify those with a QTc interval above the risk threshold for developing an arrhythmia [28,29,30]. Prolongation of the QTc interval is evident in 10–15% of patients in methadone programs [30,31,32], with an apparent greater risk in those receiving high dosages (>110 mg/day) [9], although the relationship between methadone dosage and the length of the QT interval has not been previously shown [33,34,35]. In this pilot study, there were six individuals in the control group and three individuals in the pharmacogenetic group with QTc intervals higher than the threshold that is considered dangerous (>450 ms). However, no relationship was found between the QTc interval and the methadone dosage. Two interesting findings related to the activity of the CYP2B6 enzyme, which is the enzyme responsible for the metabolism of the most cardiotoxic form of methadone (S-methadone), are worth mentioning: (i) in the group that was genotyped, the three individuals with prolonged QTc intervals showed deficient activity of the enzyme, and (ii) the average QTc was 13 ms longer in individuals with deficient activity of the enzyme than in those with normal enzyme function, although this difference was not significant (*p* = 0.33). These findings led us to suspect that the arrhythmogenic activity of methadone is not dependent on methadone dosage, but rather on the *CYP2B6* gene, as has been previously suggested by other authors [14,36,37,38].

In rehabilitation programs, methadone is the drug most commonly used as a heroin substitute. Nevertheless, effective and safe methadone doses vary significantly between individuals. Consequently, when adjusting dosages, there is a risk of provoking toxicity or delaying the perception of benefit in a patient. Medications with these characteristics are of pharmacogenetic interest. It is known that the CYP2B6 enzyme plays a dominant role in metabolizing methadone, and that the 516T allele is associated with lower expression of the enzyme, reduced catalytic activity, poor methadone metabolism, and non-response in methadone maintenance treatment compared with the native form [7,9,16,17,38,39,40,41]. However, there is limited information on the effects of *CYP2B6* genetic polymorphisms on methadone metabolism, especially in minority populations. Various *CYP2B6* alleles have been associated with changes in methadone metabolism [42]. One of the earliest identified allelic variants, CYP2B6*6, has been associated with reduced methadone metabolism [43]. In this pilot study, we confirmed that the 516G>T polymorphism (rs3745274) of the *CYP2B6* gene affects the methadone maintenance dosage (Table 5), as well as the consumption of heroin, in patients in the MMT program (Table 3). Some studies have reported that a lower methadone dose is required to stabilize patients in the MMT who are homozygotes for variant alleles of *CYP2B6*, including the allelic variant *4 (516G>T) [44,45] that was evaluated in this study (we wanted to confirm this in our population). A recent study suggested that in addition to the *CYP2B6* genotype, sex and body mass index should be included as predictors of response in methadone-dosing algorithms [40]. It is essential to mention that reports in the Caucasian population have not found differences between *CYP2B6* genotypes and the efficacy of methadone doses [46,47]. These contradictory findings might be explained by differences in the study design and the ethnicity of the population [11]. It is essential to indicate that *CYP3A4* was not genotyped because the *CYP3A4* polymorphisms leading to altered CYP3A4 function are rare, and the enzyme is more prone to inhibition and induction by co-medication and food. In addition, several studies have reported no significant associations between SNPs of *CYP3A4* and methadone dose in different populations [44,47,48]. If we take into account that the genotyped group consumed an average of 10 mg less of methadone per day after the second week of treatment (Table 5), then the cost savings, without sacrificing benefits, would justify the inclusion of the genotype (with a cost of 60 US dollars per person) in treatment protocols for heroin-addicted patients.

The WHOQOL-BREF proposed by the World Health Organization to evaluate quality of life has been previously applied and validated as a reliable instrument for measuring the quality of life among heroin-addicted patients currently treated with methadone. Although the instrument was not used in this study when the patients entered the MMT, given the unfortunate conditions in which they began the program, the eighth week of treatment clearly showed that the patients’ quality of life was not as deplorable as it may have been when starting rehabilitation. The worst scoring dominion for the patients was interpersonal relationships, consistent with the findings of other studies [49,50,51]. However, it remains concerning that the highest dropout rates in MMT programs exist among socially isolated individuals. In conclusion, the results of this study suggest that the allelic variant *4 (516G>T) of the *CYP2B6* gene may indicate a reduction in the effective dose of methadone and treatment costs in the studied population.

It is important to mention that although continued drug abuse by heroin-addicted individuals before and during the MMT is relatively frequent and impacts whether or not an MMT program is effective [52,53], in our study, a co-dependency on cocaine and/or marijuana did not affect the rates of methadone abandonment.

Finally, in a forensic context, pharmacogenetics can assist in the interpretation of drug-related deaths, especially accidental drug poisonings or cases of sudden death or suicide. In this context, pharmacogenetics is emerging as an important tool to interpret toxicological information in forensic medicine. The most investigated genes that have been shown to correlate to methadone efficacy are *CYP2C19*, *CYP2D6*, and *CYP3A4* [54]. In addition, Pharmacogenetic studies are necessary in order to evaluate the forensic and toxicological implications of the abuse of medical psychotropics or alcohol-drug interactions [55].

## 5. Limitations of the Study

Although this pilot study clarifies the effect of the *CYP2B6* polymorphism on the clinical effects of methadone in the studied population, it is necessary to highlight some limitations. First, the sample size limited the potency of the research. Second, much of the data was obtained via patient self-reporting; consequently, there are concerns regarding credibility. Third, a period of 12 weeks of observation is a very short amount of time to draw definitive conclusions in programs requiring patient care for months or even years. Fourth, the serum methadone levels were not evaluated; however, in a previous study, we reported that blood concentrations of racemic methadone and its enantiomers were significantly associated with the dose/day of the medication, but none of the demographic, clinical, or genetic variables impacted the serum levels of methadone in a similar population [10]. Other potentially influential genetic markers were not included in the study, and we do not know whether the control group has the same allelic distribution as the pharmacogenetics group. Finally, we did not obtain information concerning the use of drugs with potential pharmacological interactions with methadone that could explain some of the results obtained.

## Figures and Tables

**Table 1:** Primers for amplification of fragments and probes for genotyping.

Gene	SNP	Forward (5′-3′)	Reverse (5′-3′)	SBE (5′-3′)
*ABCB1*	rs1045642	TATGTTGGCCTCCTTTGCTG	GCTGAGAACATTGCCTATGG	ATGACGTTGAGTGGCCAATCACCTGTTCA
*CYP2B6*	rs3745274	TCGGTCTGCCCATCTATAAA	TGATTCTTCACATGTCTGCG	ATGACGTTGGATGACGTTGGATGGTTGGATTGTCTTGCTGGCACCCAAT
*OPRM1*	rs1799971	GGGTCAACTTGTCCCACTTA	TGATGGCCGTGATCATGGAG	GATGACGTTGGATGACGTTGAGTGGACTTCGGGATGGGA

**Table 2:** Baseline characteristics of patients in the conventional and pharmacogenetic group.

Characteristic	Conventional Group (n = 34)	Pharmacogenetic Group (n = 38)	*p*-Value
Age (years)	24.6 ± 5	23 ± 4	0.15
Gender (male/female)	29/5	34/4	0.59
Body mass index	20.4 ± 2.3	20.2 ± 2.1	0.61
Creatinine	1.0 ± 0.15	1.0 ± 0.12	0.85
Transaminases:			
Glutamic-pyruvic	31 ± 20	38 ± 32	0.32
Glutamic-oxaloacetic	33 ± 20	38 ± 29	0.33
Schooling			
Low education	1	2	0.57
Medium education	30	30	
Higher education	3	6	
Marital status			
Single or separated	32	34	0.48
Married or cohabiting	2	4	
Cohabitation			
Live alone	2	2	0.91
Live with family	32	36	
Employment (yes/no)	10/24	12/36	0.84
Psychiatric history (yes/no)	6/28	11/27	0.26
Current mental illness (yes/no)	1/33	3/35	0.36
Anxiety at the start (yes/no)	18/16	12/26	0.18
Criminal history (yes/no)	26/8	29/9	0.99
Age of first heroin use (years)	19.3 ± 4.9	18.1 ± 3.5	0.23
Route of administration of heroin:			
Inhaled	19	20	0.78
Intravenous	15	18	
Previous methadone treatments	0.5 ± 0.7	0.6 ± 0.8	0.47
Family history of abuse (yes/no)	29/5	27/11	0.15
Cocaine dependence (yes/no)	15/19	12/26	0.27
Cannabis dependence (yes/no)	25/9	26/12	0.63
Nicotine dependence (yes/no)	31/3	34/4	0.81

**Table 3:** Percentages of individual users of heroin during MMT.

Observation Times	Conventional Group (n = 34)	Pharmacogenetic Group (n = 38)	*p*-Value
Week 2	47.1	39.5	0.52
Week 3	45.2	34.4	0.38
Week 4	33.3	36.7	0.79
Week 5	28.6	17.9	0.34
Week 7	30.8	14.8	0.17
Week 9	34.6	14.8	0.09
Week 12	16.0	8.0	0.38

**Table 4:** Percentages of heroin addicts and marijuana and/or cocaine/crack users under MMT.

Observation Times	Conventional Group (n = 34)	Pharmacogenetic Group (n = 38)	*p*-Value
**Week 0**			
Marijuana	73.5	68.4	0.63
Cocaine/crack	44.1	31.6	0.27
**Week 2**			
Marijuana	65.6	64.9	0.95
Cocaine/crack	15.6	13.9	0.84
**Week 5**			
Marijuana	63.0	66.7	0.78
Cocaine/crack	11.1	10.7	0.96
**Week 7**			
Marijuana	64.0	69.2	0.69
Cocaine/crack	12.0	14.8	0.77
**Week 9**			
Marijuana	68.0	80.8	0.29
Cocaine/crack	20.0	7.4	0.28

**Table 5:** Methadone maintenance doses (mg/day) according to the 516G>T (rs3745274) genotype of the *CYP2B6* gene.

Observation Times	Homozygous	Allele Carrier T	*p*-Value
	GG	GT + TT	
Week 2	63 ± 8	50 ± 12	0.001
Week 3	62 ± 9	49 ± 11	0.002
Week 4	62 ± 9	49 ± 12	0.005
Week 5	62 ± 9	54 ± 8	0.024
Week 7	63 ± 11	54 ± 8	0.022
Week 9	61 ± 13	52 ± 10	0.050
Week 12	61 ± 14	51 ± 10	0.050

## Data Availability

The original contributions presented in the study are included in the article, and further inquiries can be directed to the corresponding author.
